# School bullying victimization and post-traumatic stress symptoms in adolescents: the mediating roles of feelings of insecurity and self-disclosure

**DOI:** 10.1186/s40359-023-01065-x

**Published:** 2023-01-31

**Authors:** Yongyong Xu, Yingying Ye, Yichang Zha, Rui Zhen, Xiao Zhou

**Affiliations:** 1 Department of Psychology and Behavioral Sciences, Zhejiang University, 310028 Hangzhou, China; 2 Jing Hengyi School of Education, Hangzhou Normal University, Hangzhou, 311121 China

**Keywords:** School bullying victimization, PTSSs, Feelings of insecurity, Self-disclosure

## Abstract

**Background:**

Posttraumatic stress symptoms (PTSSs) is known to be a typical problem for school bullying victims. However, the underlying mechanism between school bullying victimization and PTSSs remains unknown. This study examined the mediating roles of feelings of insecurity and self-disclosure in the relationship between school bullying victimization and PTSSs in adolescents.

**Methods:**

Self-report questionnaires were used to assess 5013 adolescents; 443 of them with school bullying experiences were selected to carry out this study.

**Results:**

The results found that school bullying victimization was directly related to more PTSSs, indirectly associated with more PTSSs through feelings of insecurity, and through the path from feelings of insecurity to self-disclosure. However, school bullying victimization did not exert an indirect effect on PTSSs through one step effect of self-disclosure.

**Conclusions:**

These findings indicated that feelings of insecurity and self-disclosure mediated the relationship between school bullying victimization and PTSSs. The psychological service should reduce adolescents’ feelings of insecurity and give them more chances to disclose their bullying experiences.

## Introduction

Bullying is considered to be an intentional and repeated physical or psychological harm that occurs between individuals of imbalanced power [1, 2], and bullying in school includes behaviors such as teasing, rumor-mongering, isolation, and beatings among peers [3], which is common in various countries [4], and its incidence rate is even as high as 30% [5–7]. As an interpersonal trauma event, school bullying not only causes physical harm to the victim, but also leads to many psychological problems [4, 8–10], among which post-traumatic stress symptoms (PTSSs) is one of the typical problems of bullying victims [11, 12]. For example, Ossa et al. have found that about 20% of individuals experienced severe school bullying during their school days, and 50% of them developed PTSSs [13], which can persist for up to many years [14]. Empirical studies also found that school bullying experience can significantly and positively predict PTSSs [13, 15–17].

While PTSSs was a common negative reaction in adolescents who suffered from school bullying, a question was raised why do these school bullying victims develop PTSSs? According to Janoff-Bulman’s hypothesis of shattered assumptions [18], traumatic events have an impact on the individual’s beliefs about the world. It can destroy the individual’s belief which assumes the world is safe and no one will be harmful, leading to the emergence of cognitive beliefs that the world is dangerous and unsafe, which may reduce their feelings of security [19], inducing their feelings of insecurity. This may reduce the initiative and enthusiasm of individuals in interpersonal communication, make it difficult to establish healthy interpersonal relationships, obtain support and help from others, and is not conducive to relying on the strength of others to relieve their own psychological problems [20], so as may cause or exacerbate PTSSs. Based on this, it can be hypothesized that as a type of interpersonal trauma, school bullying victimization could induce PTSSs by increasing feelings of insecurity.

Moreover, the shattered assumptions of Janoff–Bulman also emphasize that after traumatic events challenge people’s stable belief systems, they will also induce imbalances in individual belief systems before and after trauma. To avoid the negative effects of this imbalance, individuals may also adopt defensive measures (e.g., repression and denial) to maintain cognitive balance [21], buffering the individual’s perception of traumatic experiences and exposure to related emotions. Thus, following the traumatic experiences, some traumatized individuals do not want to disclose it. Empirical studies have also found that school bullying experience negatively correlates with victims’ self-disclosure [22]. Furthermore, self-disclosure naturally is a form of social exchange and a necessary condition for the formation and development of good interpersonal relationships [23, 24], it can also bring a certain degree of social support to individuals [25], helping them relieve stress and negative emotions [26], finally PTSSs. In addition, sharing traumatic experiences and related emotions with others is also a process of active construction, which helps individuals re-understand their own experiences and emotions from a positive perspective [27], thereby it can also alleviate the severity of PTSSs. In light of this, we hypothesized that school bullying experience might indirectly positively predict PTSSs by reducing the victim’s self-disclosure level.

Theoretically, while both feelings of insecurity and self-disclosure may mediate between school bullying victimization and PTSSs, is there a relationship between feelings of insecurity and self-disclosure? In fact, individuals' lack of security may trigger their own defense mechanisms that can hinder the expression of their own experiences and emotions. That is, individuals with high insecurity are more likely to be unsociable, hide their true feelings and thoughts, and be reluctant to self-disclose [28]. Empirical studies have also shown that individuals with high feelings of insecurity have relatively low frequencies of self-disclosure [29]. In such as case, it was suggested that feelings of insecurity could negatively predict self-disclosure.

By reviewing the previous studies, we found that although the studies on the relationship between school bullying victimization and PTSSs have been abundant, few empirical studies have focused on the mediating roles of feelings of insecurity and self-disclosure in it. Furthermore, the relationship between feelings of insecurity and self-disclosure is rarely assessed in the predictive process of bullying victimization on PTSSs. To fill these gaps, the aim of this study was to examine the mediating roles of feelings of insecurity and self-disclosure in the relationship between bullying victimization and PTSSs, and hypothesized that the feelings of insecurity and self-disclosure play multiple mediating roles in school bullying victimization and PTSSs, according to shattered assumption and existing studies. On the one hand, this study may inform the importance of interpersonal problems on psychological outcomes after bullying victimization, because feelings of insecurity and self-disclosure were considered as important aspects of interpersonal relationships following school bullying experiences. On the other hand, this may also provide psychological interventions for bullied adolescents with a specific target that focuses on improving interpersonal relationships, particularly increasing their feelings of interpersonal safety and self-disclosure. Moreover, this study provided a new perspective for clinical intervention on psychological problems of adolescents with bullying victimization.

## Methods

### Participants and procedures

This study used convenience sampling to recruit participants. We aimed to recruit a relatively large sample to better understand the situation of school bullying victimization among adolescents. In 2019, we firstly contacted school principals of two senior high schools we knew in Anhui Province, China, and informed them the aim of this investigation. After obtaining their approval, we recruited students in all the classes from Grades 10 and 11 who attended school on the investigation day. Students in Grade 12 were not recruited because of their busy academic schedules. We printed a total of 5500 questionnaires, mailed them to these schools, and the teachers distributed and collected the questionnaires. In total, 5013 students participated in our paper-and-pencil investigation, of which 2410 were boys, 2450 were girls, and 153 did not fill in their sex; 2531 were in Grade 11, 2480 were in Grade 12, and 2 did not fill in their grades; the mean age at the test was 16.77 years (*SD* = 0.92 years), and the range was between 12 and 22 years, while 155 did not fill in their ages. All of the adolescents were asked not to mention their names in the questionnaire and the teachers were asked not to review the questionnaires.

A self-report Trauma Event Checklist revised from the Life Event Scale was used to screen adolescents with school bullying experiences [30]. If the participant checked the item “school bullying”, he/she is considered to have been bullied. Based on this, we found that 443 out of 5013 adolescents were bullied. In this study, we selected the data of these 443 bullied adolescents for analysis and discussion, which was perfect enough for the sample size requirement (n = 89, power = 0.95) for the linear multiple regression test, according to the G*Power result. And for the participants, 322 were boys, 106 were girls, and 15 did not fill in their sex; 228 were in Grade 11, 214 were in Grade 12, and 1 did not fill in the grade; the mean age at the test was 15.81 years (*SD* = 0.92 years), and the range was between 13 and 19 years, while 14 did not fill in their ages.

This study was approved by the Research Ethics Committee of the School of Medicine, Zhejiang University (No. 2019-051). The participation of participants is completely voluntary, and they can choose to withdraw from the study at any time. Informed consent was obtained from their guardians. All procedures performed in this study involving human participants were in accordance with the ethical standards of the research committee. No compensation was provided for any participants. The formal investigation was administrated in a classroom setting.

### Measures

#### Delaware Bullying Victimization Scale

This study used the Chinese version of Delaware Bullying Victimization Scale to assess the school bullying victimization of adolescents [31]. The scale has a total of 17 items, divided into four sub-scales: verbal, physical, social/relational, and cyberbullying victimization, of which the 13th item is not included in the data analysis as a screening item. The items of the scale are scored on a 6-point Likert scale, where 0 means "never", and 5 means "every day", with higher scores indicating more bullying. The bullying measure has been validated for use with Chinese adolescents, and Cronbach’s alpha in the original Chinese version is 0.91. In this study, the reliability of the scale was good (Cronbach’s α = 0.91).

#### Security Questionnaire

The Security Questionnaire developed by Cong and An was used to measure the feelings of insecurity of adolescents [32], and it has been validated for use with Chinese adolescents, with a Cronbach’s alpha of 0.80. The scale has a total of 16 items and uses a 5-point score, with 1 representing "completely incompatible" and 5 representing "totally suitable". The scale has good reliability in adolescents [33]. In this study, the scale’s Cronbach’s α coefficient was 0.89.

#### Distress Disclosure Index Scale

This study used the Distress Disclosure Index Scale revised by Zhen et al. to measure adolescents’ self-disclosure [27]. The scale has a total of 12 items and is scored on a 5-point scale, where 0 means "strongly disagree" and 4 means "strongly agree". The questionnaire mainly measures the level of self-disclosure by measuring the degree to which individuals tend to tell others about their troubles, trauma, pain, and other negative information. This questionnaire showed predictive validity in the Chinese adolescent sample, and its Cronbach’s alpha is 0.60. In this study, the reliability of the scale was good, with a Cronbach’s α coefficient of 0.87.

#### PTSD Checklist for DSM-5

The self-report PTSD Checklist for DSM-5 was developed by Weathers et al. and revised by Zhou et al. [34, 35], which showed good validity in Chinese adolescents and has a Cronbach’s alpha of 0.90. This 20-item self-reported scale was developed to assess the occurrence and frequency of PTSSs related to the school bullying victimization experiences. The scale has the four following subscales: intrusions, negative cognition and emotion alteration, avoidance, and hyperarousal. In the present study, all respondents rated the frequency of symptoms during the last 2 weeks on a 5-point scale that ranged from 0 (“not at all/only once”) to 4 (“almost every day”). In this study, the overall Cronbach’s α coefficient of the scale was 0.90.

### Data analysis

Descriptive statistics and correlation analysis was performed using SPSS19.0, and Mplus 8.0 software was used to build a multiple mediation model. To evaluate the model fit, we used chi-square values, the comparative fit index (CFI), Tucker–Lewis index (TLI), root mean square error of approximation (RMSEA), and standardized root means square residual (SRMR). The general cutoffs for model acceptance were ≥ 0.90 for the CFI and TLI, and < 0.08 for the SRMR and RMSEA. Missing data was handled with the maximum likelihood estimation in the model.

Based on the hypothesis, we first examined a direct effect model with school bullying victimization predicting PTSSs. Based on the direct effect model, we inserted the feelings of insecurity and self-disclosure in the relationship between school bullying victimization and PTSSs, and thus built a multiple indirect effect model. Next, we performed bias-corrected bootstrap tests with 95% confidence intervals (CIs) to evaluate the significance levels of the indirect effects observed in the multiple mediation model.

## Results

### Descriptive statistics and correlation between main measures

Table 1 shows the descriptive statistics and correlation analysis on the school bullying victimization, feelings of insecurity, self-disclosure, and PTSSs. The results demonstrated that school bullying victimization was significantly and positively related to feelings of insecurity and PTSSs, respectively, while the correlation between school bullying victimization and self-disclosure was not significant. The feelings of insecurity was significantly and negatively related to self-disclosure and positively related to PTSSs. There was a significant negative correlation between self-disclosure and PTSSs.

**Table1:** Correlations among main variables

	*M* (*SD*)	1	2	3
1. School bullying victimization	10.88 (11.51)	–		
2. Feelings of insecurity	47.86 (11.56)	.277**	–	
3. Self-disclosure	23.31 (8.71)	− .008	− .331**	–
4. PTSSs	39.77 (14.14)	.323**	.653**	− .274**

**p < .01

### Examination of the mediating effects

As the description of the data analysis section, we first examined the direct relationship between school bullying victimization and PTSSs by building a direct effect model. The results found that this model fitted data completely, *χ*^2^(0) = 0.000, CFI = 1.000, TLI = 1.000, RMSEA(90%CI) = 0.000, SRMR = 0.000. The path results found that school bullying victimization positively predicted PTSSs (*β* = 0.397, *p* < 0.001).

**Fig. 1:**
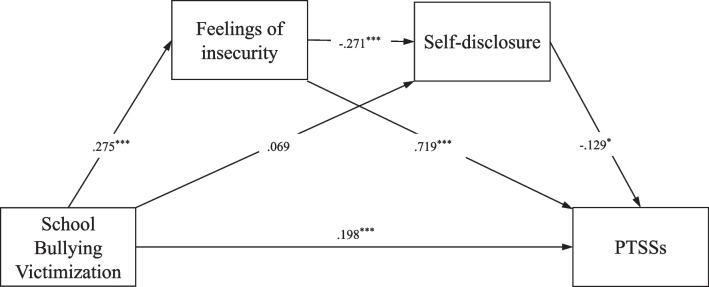
The multiple mediation model. Notes: *p < .05, **p < .01, ***p < .001

Based on the direct effect model, we built a multiple mediation model (see Fig. 1). It was found that the model perfectly fitted the data, *χ*^2^(0) = 0.000, CFI = 1.000, TLI = 1.000, RMSEA (90% CI) = 0.000, SRMR = 0.000. The results of path analysis found that school bullying victimization can significantly positively predict feelings of insecurity and PTSSs, but has a non-significant prediction on self-disclosure; The feelings of insecurity significantly negatively predicted self-disclosure and positively predicted PTSSs, and self-disclosure significantly negatively predicted PTSSs. These results indicate that school bullying victimization can directly and positively predict PTSSs, indirectly and positively predict PTSSs through feelings of insecurity, and through feelings of insecurity via self-disclosure. However, school bullying victimization did not indirectly exert a significant predictive effect on PTSSs through self-disclosure.

Next, we conducted the bias-corrected bootstrap test with a 95% CI to examine the significance of the above-mentioned mediating effect, and the results are shown in Table 2. It can be seen from Table 2 that except for the 95% CI [− 0.032 to 0.000] of the indirect path from school bullying victimization to PTSSs via self-disclosure, the rest of the indirect paths do not include 0. These findings prove that the above mediating effect is established, among which the mediation effect value was 0.199, accounting for 50% of the total effect [0.199/0.397].

**Table2:** Bias-corrected Bootstrap tests of mediating effects

Paths from school bullying victimization to PTSSs	95% CI	*β*
Direct path	[.130–.286]	.199
Indirect path via feelings of insecurity	[.131–.282]	.198
Indirect path via self-disclosure	[− .032 to .000]	− .009
Indirect path via feelings of insecurity and self-disclosure	[.001–.026]	.010

A 95% CI of an indirect path coefficient that does not include 0 suggests that the indirect path is significant

## Discussion

This study examined the mechanism underlying the relation of school bullying victimization to PTSSs through feelings of insecurity and self-disclosure in adolescents. The findings supported the shattered assumption and suggested interpersonal trauma might be an important risk factor for PTSSs. Furthermore, this study also found a deep mechanism underlying bullying victimization predicting PTSSs by integrating the feelings of insecurity and self-disclosure, which shed light on building a larger framework for PTSSs following interpersonal trauma. It also enlightens how we can help adolescents to protect themselves from the negative reactions following bullying experiences.

The finding that school bullying victimization directly predicted PTSSs supports the previous findings [13, 17] and suggests that individuals who suffered from school bullying victimization are more likely to develop PTSSs. One possible explanation is that experience of being bullied may cause negative affectivity [36], which is linked to role conflicts [37] and interpersonal conflicts [38, 39]. Moreover, evasive behavior and strong stress arousal are two traits of bullied victims, which are also highly correlated with PTSSs [40]. In light of these, school bullying victimization was related to more PTSSs.

We also found that school bullying victimization had an indirect effect on PTSSs through feelings of insecurity. The possible reason for this is that after undergoing school bullying, teenagers may suspect the security of the world they are living in and thus their feelings of insecurity will increase. It may cause them to defend themselves in an unsafe environment and reducing communication with others may be an effective way for them, which then hurt their interpersonal relationship [19]. This further will reduce the possibility of looking for peer’s help so as decrease the social support teenagers can get [20]. Because social support is an important psychosocial resource, the reduction of social support informs the loss of resources, and thus may elicit a series of negative psychological reactions including PTSSs, according to the conservation of resources theory [41].

We also found that school bullying victimization can positively predict PTSSs through the multiple mediating effects of feelings of insecurity via self-disclosure. In fact, the feelings of insecurity were found to be increased following school bullying experiences as the discussion in the paragraph above. However, studies had illustrated that feelings of insecurity were related to more alexithymia [42, 43], which refers to individuals who avoid close interpersonal relationships and keep distant from others socially [44], and thus individuals with high levels of insecurity resist self-disclosure [29]. Self-disclosure is helpful for individuals to strengthen their bond and intimacy with others [45], and thus increase the social cohesion of communities [46, 47], this may activate communal coping [48], finally relieving posttraumatic psychological distress [27]. However, once the individual being resistant to disclosure, then they did not form effective coping, and finally might lead to more PTSSs.

In contrast with our hypothesis and a previous study [22], this study found that school bullying did not significantly predict PTSSs through self-disclosure. A possible reason for this is that most of the participants of our study are left-behind children. Considering their special identities, we can imagine that due to the lack of parents’ company, these students themselves lack the objects with whom they can take in-depth communication, so the degree of self-disclosure is at a low level for them all. Therefore, whether they have been bullied at school cannot cause a significant influence on their self-disclosure. In addition to this explanation, other rationales should be further examined in future study.

This study has some limitations that should be noted. First, our study was a cross-sectional design, and thus the causal relation should be made cautiously. Second, we only examined adolescents’ school bullying victimization on PTSSs, and other traumatic experiences in these adolescents did not be considered, and whether these experiences may exert extra effects on the current findings was unknown. Third, this study used convenience sampling but not stratified random sampling technique to select two high school students in Anhui Province, China, so whether the results can be applied to other groups from different ages or cultural contexts needs further examination. Larger participants would be selected by the stratified random sampling technique in the future study. Fourth, this study only assessed PTSSs on a symptom level, and did not compare the possible differences between the clinical PTSSs group and the non-clinical PTSSs group. Nevertheless, our study integrated existent theories and findings to elucidate the deep mechanism underlying the relation of school bullying victimization to PTSSs by feelings of insecurity and self-disclosure. These findings also provide clinicians with insights into treatments and interventions, wherein increasing the feelings of security and giving more chances for them to express themselves can also help adolescents with bullying experiences to recover from PTSSs.

## Declarations

### Availability of data and materials

The datasets generated and analysed during the current study are not publicly available, but are available from the corresponding author upon reasonable request. The reason is that these datasets belong to our lab and we are still trying to analyze them for further discoveries.
